# Clinical and Molecular Characterization of a Rare EGFR cis Compound L833V/H835L Mutation in Non–Small Cell Lung Cancer

**DOI:** 10.1158/2767-9764.CRC-25-0831

**Published:** 2026-04-22

**Authors:** Yo Han Jeon, Ahjin Lim, Myung Kyung Choi, Hee Seong Choi, Yurimi Lee, Hyunju Song, Hyun-Soo Cho, Jeonghee Cho, Yoon-La Choi

**Affiliations:** 1 Department of Pathology, Kosin University College of Medicine, Busan, South Korea.; 2 Department of Biomedical Science and Biosystems, Dankook University, Cheonan, South Korea.; 3 Department of Nanobiomedical Science, Dankook University, Cheonan, South Korea.; 4 International Consortium for Development of Innovative Anti-Cancer Drugs (ICD^2^), Cheonan, South Korea.; 5 Department of Systems Biology, College of Life Science and Biotechnology, Yonsei University, Seoul, South Korea.; 6 Department of Pathology and Translational Genomics, Samsung Medical Center, Sungkyunkwan University School of Medicine, Seoul, South Korea.; 7 Department of Health Sciences and Technology, SAIHST, Sungkyunkwan University, Seoul, South Korea.

## Abstract

Non–small cell lung cancer (NSCLC) with *EGFR* L833V/H835L in cis mutation remains poorly understood. We retrospectively reviewed the clinicopathologic features of NSCLC with either *EGFR* L833V or H835L mutation. The study population includes patients with NSCLC (*n* = 3,835) at Samsung Medical Center, Seoul, Korea. A total of eight patients with *EGFR* L833V/H835L in cis mutation were identified. Poorly differentiated histology (either micropapillary or solid patterns) presented in four of five resected cases. Four patients received EGFR tyrosine kinase inhibitors (TKIs) and achieved a median progression-free survival of 13 months. The oncogenic and therapeutic properties of the mutation were investigated through a combination of functional and biochemical analyses, utilizing both *in vitro* and *in vivo* models, and structural modeling. The L833V/H835L mutant exhibited oncogenic potential, transforming NIH-3T3 cells and promoting IL3-independent growth in Ba/F3 cells. Furthermore, EGFR TKIs effectively suppressed the mutant’s oncogenic activity in both *in vitro* and *in vivo* studies. These phenomena are further supported by its increased kinase activity in structural modeling. Although rare, *EGFR* L833V/H835L in cis mutation represents a biologically oncogenic and clinically actionable variant in NSCLC.

**Significance:**

Our study reveals the rare *EGFR* L833V/H835L cis mutation as an aggressive oncogenic driver in lung adenocarcinoma, highly responsive to EGFR-directed therapy. These findings provide crucial therapeutic strategies for patients with this specific *EGFR* variant, addressing a current gap in precision medicine.

## Introduction

Somatic mutations in Epidermal growth factor receptor (*EGFR*) tyrosine kinase domain are the most common driver mutations of non–small cell lung cancer (NSCLC) in East Asia and are detected in about 60% of lung adenocarcinomas from East Asian never-smoker female patients (1, 2). As advanced sequencing technologies like next-generation sequencing (NGS) are implemented in real-world clinical practice, cases with the co-occurrence of two or more mutations in *EGFR* kinase domain have been reported, the majority of which are coexisting of one classic sensitizing *EGFR* mutation and one or more rare mutation (3, 4). However, the clinical significance of identified rare mutations, especially when two rare *EGFR* mutations are present in cis, remains largely unknown. Regarding *EGFR* L833V/H835L mutations in cis, only a handful of documented cases without histopathologic analysis have been reported (Supplementary Table S1; refs. 5–16). Therefore, characterizing their oncogenic potential is crucial for subsequent clinical application with existing *EGFR*-targeted drugs.

In this study, we retrospectively investigated the clinicopathologic characteristics of NSCLC harboring the rare *EGFR* L833V/H835L mutations. In addition, we systematically compared the functional and biochemical features of the L833V/H835L mutant with those of single mutations. The clinical significance of L833V/H835L mutant was further determined by integrating drug response analysis utilizing L833V/H835L mutant–driven cell models, complemented by structural modeling.

## Materials and Methods

### Study population

Study population includes patients with NSCLC who underwent either Sanger sequencing analysis of *EGFR* exons 18 to 21 (*n* = 2,701, between January 2009 and August 2012), of which the positive rate was 33.06% (*n* = 893), or NGS analysis using TruSight Oncology 500 (TSO500, Illumina) panels (*n* = 1,134, between January 2021 and October 2023) at Samsung Medical Center in Seoul, Korea. Patients with NSCLC with either *EGFR* c.2497T>G (p.Leu833Val) mutation in exon 21 or *EGFR* c.2504A>T (p.His835Leu) mutation in exon 21 were finally included in this study. Patients with NSCLC who underwent PCR-based *EGFR* tests only (cobas EGFR Mutation Test v2 and GenesWell ddEGFR Mutation Test) were excluded from the population as those tests were not able to capture either *EGFR* L833V or H835L mutation.

### Data collection and review of histopathology

Clinical information including age at diagnosis, sex, smoking status, stage at initial diagnosis, pathologic diagnosis, molecular testing results for *EGFR* mutations, and clinical follow-up data were retrieved from electro medical records. All available pathology slides were reviewed by three pathologists (Y.H. Jeon, Y. Lee, and Y.-L. Choi). Tumor stage was defined according to the eighth edition of the American Joint Committee on Cancer. The following definitions were used for the histologic assessment of surgically resected lung adenocarcinoma. Histologic pattern occupying more than 10% of the tumor area was recorded. The predominant pattern was defined as the pattern that covers the largest portion of the tumor area. Tumors with lepidic predominance and with no or less than 20% of high-grade patterns (solid, micropapillary, or complex glandular pattern) were defined as well-differentiated tumors, and tumors with acinar or papillary predominance with no or less than 20% of high-grade patterns as moderately differentiated tumors. Tumors with 20% or more of high-grade were defined as poorly differentiated tumors (17). This study was approved by the Samsung Medical Center Institutional Review Board (IRB No. 2023-11-148) and was conducted in accordance with the Declaration of Helsinki. As this study was a retrospective study, the requirement for informed consent was waived.

### Structure prediction

Structures of *EGFR* kinase domain variants (L833V, H835L, and L833V/H835L), including dimeric assemblies, were predicted using AlphaFold3 (18). Amino acid sequences of the target proteins were input into the AlphaFold3 pipeline without template structures to enable *de novo* predictions. Model confidence was assessed based on the predicted local distance difference test scores. Inhibitor-bound complex structures were also predicted using AlphaFold3, with both the amino acid sequences and Simplified Molecular Input Line Entry System (SMILES) representations of the ligands provided as input. All structural analyses and visualizations were performed using PyMOL (Schrödinger; RRID: SCR_000305).

### Expression and purification of EGFR kinases

The human *EGFR* kinase domains (residue 696–1,022) were cloned into the pFastBac1 vector (RRID: Addgene_1956) as a fusion with N-terminal 10x His tag and tobacco etch virus (TEV) protease cleavage site. Wild-type (WT) *EGFR* kinase, as well as L833V, H835L, L833V/H835L and L858R mutants, were generated by site-directed mutagenesis. *EGFR* kinase domain was expressed in *Spodoptera frugiperda* (Sf9) cells (2 × 10^6^ cells/mL) and lysed by sonication in lysis buffer (300 mmol/L NaCl, 25 mmol/L Tris pH 7.5, 30 mmol/L imidazole, and 10% glycerol) with 0.5 mmol/L tris(2-carboxyethyl) phosphine hydrochloride (TCEP) and protease inhibitor cocktail. The supernatant fraction was isolated by centrifugation at 13,000 rpm at 4°C for 1 hour. The protein was incubated at 4°C for 2 hours with nickel-affinity resin and eluted with elution buffer (300 mmol/L NaCl, 25 mmol/L Tris-HCl pH 7.5, 10% glycerol, and 300 mmol/L imidazole) with 0.5 mmol/L TCEP. After overnight cleavage with TEV protease, the sample was further purified by size-exclusion chromatography (SEC) on Superdex 200 increase (Cytiva) with SEC buffer (20 mmol/L Tris-HCl pH 7.5, 300 mmol/L NaCl, and 0.5 mmol/L TCEP). Each protein was concentrated, and aliquots were stored at −80°C until kinase analysis.

### *In vitro* kinase assay

EGFR kinase activities were measured using the Kinase-Glo Luminescent Kinase Assay Kit (Promega) according to the manufacturer’s instructions. The kinase reactions were performed using reaction buffers containing 40 mmol/L Tris-HCl pH 7.5, 20 mmol/L MgCl_2_, 0.1 mg/mL BSA, 2 mmol/L MnCl_2_, and 1 mmol/L TCEP in 96-well white plates (SPL). Relative activity assays were performed in a total volume of 50 μL containing 500 ng EGFR kinase domain proteins, 10 μmol/L ATP, and 0.2 mg/mL poly-Glu-Tyr 4:1 peptide (Sigma, ; RRID: SCR_000488) as a substrate. The kinase reaction was incubated for 1 hour at room temperature, and 50 μL of Kinase-Glo reagent was added to stop the kinase reaction. The remaining ATP was reacted with luciferase and further incubated for 10 minutes at room temperature to stabilize the luminescent signal. EGFR kinase inhibition assays were performed in the same manner. EGFR kinase proteins were preincubated with increasing concentrations (0–250 nmol/L) of erlotinib, afatinib, or osimertinib for 10 minutes at room temperature, followed by the addition of ATP and peptide substrate. All inhibitors were dissolved in dimethyl sulfoxide (DMSO), and the final reaction mixtures contained 0.1% DMSO. The luminescence was measured on a luminometer (CLARIOstar Plus multilabel plate reader, BMG LABTECH). All kinetic data were calculated using GraphPad Prism software version 7.0 (RRID: SCR_002798).

### Expression constructs

WT and L858R-mutant EGFR expression plasmids were previously described (19). L833V, H835L, and L833V/H835L mutant EGFR expression plasmids were generated from a WT EGFR in pBabe-puro (RRID: Addgene_11011) template using QuikChange site-directed mutagenesis (Agilent Technology), following the manufacturer’s protocol.

### Cell culture generation of cell lines by retroviral transduction and reagents

NIH-3T3 cells were purchased from the Korean Cell Line Bank and Ba/F3 cell was kindly provided by Dr. Matthew Meyerson. NIH-3T3 and Ba/F3 cells were cultured according to standard ATCC protocols or as previously established methods (20). All cell lines were authenticated by short tandem repeat profiling at the time of acquisition, and no additional authentication was performed after the cells were received in our laboratory. Stable cell lines of both NIH-3T3 (RRID: CVCL_0594) and Ba/F3, expressing either WT or mutant EGFR, were created through retroviral infections and pooled, as detailed in reference (19). Before EGF stimulation and subsequent harvesting, all cultures were subjected to an 18-hour period of serum starvation. EGF (Invitrogen) was applied at a concentration of 25 ng/mL for 5 minutes for stimulation, with deviations noted only when necessary. Erlotinib, afatinib, and osimertinib were purchased from Chemscene and dissolved in DMSO. All experiments were performed with *Mycoplasma*-free cells. For all experiments, cells were used within 50 passages after thawing.

### Immunoblotting and antibodies

Cells were lysed in RIPA buffer (50 mmol/L Tris-HCl, pH 7.4, 150 mmol/L NaCl, 5 mmol/L EDTA, 1% NP-40, 0.5% sodium deoxycholate, and 0.1% SDS) supplemented with sodium orthovanadate (0.2 mmol/L), BGP (0.2 mmol/L), aprotinin (0.5 μg/mL), leupeptin (0.5 μg/mL), and PMSF (1 mmol/L). For immunoblotting, 50 to 100 μg of protein was separated by 8% to 10% SDS-PAGE, transferred, and probed with antibodies. Antibodies against anti-EGFR antibody (RRID: AB_386099; #A300-388A) was purchased from Bethyl Laboratory (; RRID: SCR_013554). p-EGFR (RRID: AB_331701; Y1068; #2234S), p-EGFR (RRID: AB_823485; Y1086; #2220S;), total AKT (RRID: AB_329827; #9272S), and p-AKT (RRID: AB_329825; Ser473; #9271S) were obtained from Cell Signaling Technology. Anti–phospho-tyrosine (4G10; #05-321; RRID: AB_2891016) was obtained from Millipore. β-Actin (RRID: AB_626630; #sc-8432) antibody was sourced from Sigma-Aldrich and Santa Cruz Biotechnology, respectively.

### Anchorage-independent growth assay

Soft agar assays were conducted in the presence or absence of EGF and/or drugs at the indicated concentrations in triplicate as previously described (21). After 2 to 3 weeks, digital images were taken, and the size of colonies was quantified using ImageJ software (NIH, RRID: SCR_003070). Data were presented as a relative ratio in a graph after normalization to relative sizes of colonies formed by control cells. Each assay was repeated a minimum of three times with comparable results.

### Xenografted mouse study

All animal experimental procedures were approved and carried out in accordance with the Institutional Animal Care and Use Committee at Dankook University (DKU-25-005) in Cheonan, Republic of Korea. Vector-transduced parental or EGFR mutant–expressing NIH-3T3 cells (RRID: CVCL_0594; 5 × 10^6^ cells/100 μL) were injected into 5-week-old female BALB/c-nude mice (RRID: IMSR_CRL:194; *n* = 40 mice per *EGFR*-mutant cell line). Mice were randomly assigned to erlotinib, afatinib, osimertinib or vehicle groups for each cell line (*n* = 9–10 mice per group). After the tumor size reached approximately 150 to 200 mm^3^, mice were treated with erlotinib (25 mg/kg), afatinib (20 mg/kg), and osimertinib (5 mg/kg) daily by oral gavage. Tumor volume was measured with a caliper daily and calculated using the formula *volume* = *length* × *width*^*2*^/*2*. Mice were sacrificed when morbid.

### IL3-independent cell proliferation assay

The *EGFR* mutants with the above mutant expressing Ba/F3 cells that grew after IL3 withdrawal for 14 days. IL3-independent cell proliferation ability of Ba/F3 cell lines were assayed by seeding (2 × 10^5^ cells/well) in a six-well plate and counting cell numbers on 15 to 21 days after IL3 withdrawal. The results were indicated means of three independent counts per well.

### Cell viability assay

After 24 hours of cell seeding in 96-well plates, the cells were treated with erlotinib, afatinib, or osimertinib at the indicated concentrations and further incubated for 3 days. The viable cells were assessed using Cell Counting Kit-8 solution (Dojindo Laboratories), and the absorbance was measured at 450 nm after 3 hours. The data were expressed as percent growth relative to untreated control cells.

## Results

### Histopathologic characterization of *EGFR* L833V and H835L mutations in patients with lung adenocarcinoma

**Figure 1.**
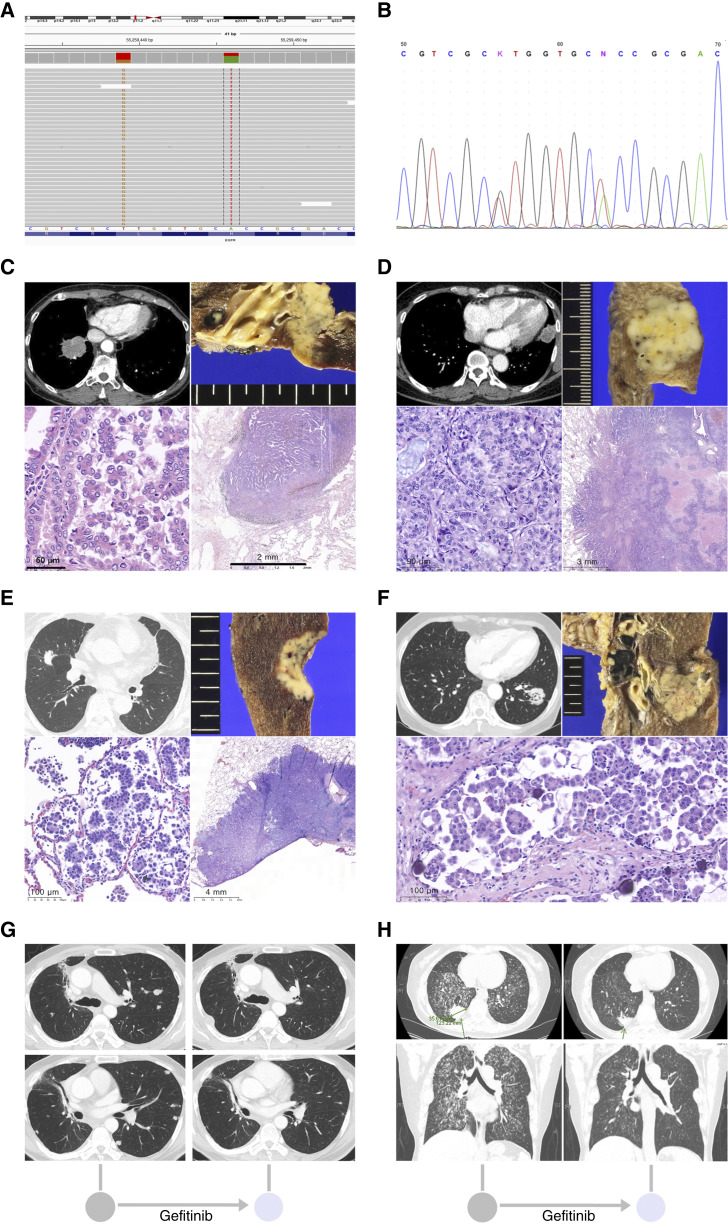
Detection of *EGFR* L833V/H835L in cis mutations and its histopathologic characteristics in the surgically resected NSCLC. **A** and **B,***EGFR* c.2497T>G (p.Leu833Val) mutation and *EGFR* c.2504A>T (p.His835Leu) mutations presenting in cis were detected by NGS analysis (**A**) of the surgical specimen from patient 8 and Sanger sequencing (**B**) of the lung biopsy specimen from patient 2. **C,** Patient 4, a 69-year-old, never-smoker female, was preoperatively evaluated as cN0. Note the gross and microscopic findings of metastatic tumors in a peribronchial lymph node. **D,** Patient 5, a 62-year-old, never-smoker female, was also preoperatively evaluated as cN0. Pathologic examination of her lobectomy specimen revealed poorly differentiated adenocarcinoma with solid pattern (50%) with PL2 pleural invasion (pT2aN0M0). **E,** Patient 6, a 74-year-old, never-smoker female. Pathologic examination showed poorly differentiated adenocarcinoma with micropapillary pattern (20%) and both N1 and N2 metastases (pT2N2M0). **F,** Patient 8, a 79-year-old, never-smoker female, received pneumonectomy of the left lung. Poorly differentiated adenocarcinoma with micropapillary pattern (30%) was identified in the histopathologic examination (pT3N2M0). **G,** Patient 1, a 53-year-old, ex-smoker male (initial stage: cT1aN2M0). Palliative 1 L gemcitabine/cisplatin obtained PFS of 3 months, whereas the 2 L gefitinib regimen obtained PFS of 17 months. **H,** Patient 2, a 51-year-old, never-smoker female, (initial stage: cT2aN0M1b, rib metastasis) received 1 L palliative pemetrexed and cisplatin for 1 month, but disease progressed with lung-to-lung metastasis. Four months after the administration of 2 L gefitinib, the extent of innumerable metastatic lung nodules decreased, and the PFS was 9 months. PCR sequencing after the disease progression revealed *EGFR* T790M mutation. The patient refused to participate in the clinical study and expired after 1 month.

A total of 11 patients with either *EGFR* L833V or H835L mutation in exon 21 were identified via PCR sequencing or NGS (Table 1). Patients 1 to 3 and 9 to 11 are cases in which *EGFR* L833V or H835L mutations were confirmed by PCR sequencing before NGS became standard in clinical practice (January 2009–August 2012). Patients 4 to 8 are cases in which *EGFR* L833V or H835L mutations were identified using NGS (January 2021–October 2023). All eight patients with *EGFR* H835L mutation had concurrent *EGFR* L833V mutation, and the compound mutation was in cis from the first molecular testing. Among eight patients with *EGFR* L833V/H835L mutations in cis, five were detected by NGS (Fig. 1A) and three were detected by Sanger sequencing (Fig. 1B). All five NGS-detected *EGFR* L833V/H835L in cis cases exhibited nearly identical variant allele frequencies, suggesting a common clonal origin. In those five NGS-detected cases, PCR-based *EGFR* tests (either cobas EGFR Mutation Test v2 or GenesWell ddEGFR Mutation Test) had been performed on the same tumor sample but could capture neither L833V nor H835L. Three patients (patients 9, 10, and 11) had one classic sensitizing *EGFR* mutation [either exon 19 deletion (Ex19del) or L858R] plus L833V mutation. The incidence of either *EGFR* L833V or H835L mutation among the *EGFR* mutation–positive population was 0.62% (6 of 972) by Sanger sequencing. Nine (81.81%) patients were never-smokers, and eight (72.73%) patients were female. All 11 patients had adenocarcinoma. Six patients (patients 2, 3, 7, 9, 10, and 11) were in stage IV at initial diagnosis. Detailed histologic review was available for the five patients who received surgical resection. Four patients (patients 4, 5, 6, and 8) received surgery without neoadjuvant chemotherapy. All four patients showed 20% or more high-grade patterns (micropapillary in three cases and solid pattern in one case; Fig. 1C–F). All four patients were evaluated as to be clinical N0 stage (cN0) or clinical N1 stage (cN1) at initial stage work-up, but pathologic examination revealed N2 lymph node metastasis.

**Table 1.** Clinicopathologic features of the NSCLC harboring either *EGFR* L833V or H835L mutation.

Patient no.	Initial *EGFR* mutation detection method^a^	Initial *EGFR* status^b^	Sex/age at initial diagnosis	Smoking history	Histologic diagnosis	Specimen type	Stage at initial diagnosis	EGFR TKI use	PFS (months)	OS (months)	Additional *EGFR* mutation test (months)^c^
**Combination of L833V and H835L**											
Patient 1	PCR sequencing	c.2497 T>G p.L833V c.2504 A>T p.H835L	M/54	Ex-smoker	ADC m/d acinar pattern	Lung, resection (after adjuvant CTx)	IIIA (cT1aN2M0)	Gefitinib (2 L)	17	49	N/A
Patient 2	PCR sequencing	c.2497 T>G p.L833V c.2504 A>T p.H835L	F/51	Never-smoker	ADC	Lung, biopsy	IVA (cT2aN0M1b, Rib)	Gefitinib (2 L)	9	10	T790M only by PNA-mediated PCR clamping, /9
Patient 3	PCR sequencing	c.2497 T>G p.L833V c.2504 A>T p.H835L	F/84	Never-smoker	ADC	Pleura, biopsy	IVA (cT1N1M1c)	Supportive care only	—	9	N/A
Patient 4	NGS	c.2497 T>G p.L833V (VAF: 17.83%) c.2504 A>T p.H835L (VAF: 17.78%)	F/69	Never-smoker	ADC p/d papillary and micropapillary (20%)	Lung, resection	IIIA (pT2bN2M0)	Adjuvant CTx other than EGFR TKI	—	>51	N/A
Patient 5	NGS	c.2497 T>G p.L833V (VAF: 43.41%) c.2504 A>T p.H835L (VAF: 43.44%)	F/62	Never-smoker	ADC p/d acinar and solid (50%)	Lung, resection	IB (pT2N0M0)	Adjuvant CTx other than EGFR TKI	—	16	N/A
Patient 6	NGS	c.2497 T>G p.L833V (VAF: 23.73%) c.2504 A>T p.H835L (VAF: 23.68%)	F/74	Never-smoker	ADC p/d papillary and micropapillary (20%)	Lung, resection	IIIA (pT2N2M0)	Adjuvant CTx other than EGFR TKI	—	>32	N/A
Patient 7	NGS	c.2497 T>G p.L833V (VAF: 4.14%) c.2504 A>T p.H835L (VAF: 3.92%)	F/65	Never-smoker	ADC	LN, 11R, biopsy	IVA (cT1bN1M1a, pleura)	Afatinib (2 L)	4	9	No mutation by Geneswell ddEGFR mutation test/4
Patient 8	NGS	c.2497 T>G p.L833V (VAF: 32.31%) c.2504 A>T p.H835L (VAF: 32.10%)	F/79	Never-smoker	ADC p/d acinar and micropapillary (30%)	Lung, resection	IIIB (pT3N2M0)	Erlotinib (2 L)	>17	>17	N/A
**Combination of L833V with either L858R or Ex19del**											
Patient 9	PCR sequencing	c.2235_2249del p.E746_A750del c.2497 T>G p.L833V	F/63	Never-smoker	ADC	Lung, biopsy	IVA (cT4N2M1b, Brain)	Gefitinib (3 L)	57	82	N/A
Patient 10	PCR sequencing	c.2573 T>G p.L858R c.2497 T>G p.L833V	M/62	Never-smoker	ADC	Lung, biopsy	IVA (cT2aN2M1b, Brain)	Gefitinib (1 L)	31	47	N/A
Patient 11	PCR sequencing	c.2573 T>G p.L858R c.2497 T>G p.L833V	M/63	Ex-smoker	ADC	Lung, biopsy	IVA (cT2N3M1b, Brain)	Erlotinib (3 L)	4	8	L858R and T790M only by cobas EGFR Mutation Test v2/4

Abbreviations: ADC, adenocarcinoma; CTx, chemotherapy; LN, lymph node; m/d, moderately differentiated; N/A, not available; p/d, poorly differentiate; VAF, variant allele frequency.

a *EGFR* mutation detection method at initial diagnosis (other than PCR-based assay).

b *EGFR* mutation status at initial diagnosis (with VAFs in NGS cases).

c Additional *EGFR* mutation test/time elapsed from EGFR TKI initiation to the test (months).

### Clinical response to EGFR-targeted therapy in EGFR L833V/H835L

Total seven patients (patients 1, 2, 7, 8, 9, 10, and 11) received first- or second-generation EGFR tyrosine kinase inhibitors (TKI; Table 1). Three patients (patients 9, 10, and 11) with *EGFR* Ex19del/L833V or L858R/L833V archived median progression-free survival (PFS) of 31 months (ranging from 4 to 57 months). Four patients with *EGFR* L833V/H835L mutations (patients 1, 2, 7, and 8) received the EGFR TKIs and showed a median PFS of 13 months (ranging from 4 to 17 months). Patients 1 and 2 (Fig. 1G and H) received the first-generation TKIs as a palliative second-line chemotherapy obtaining PFS rates of 17 and 9 months, respectively, whereas patient 7 received the second-generation TKI as a palliative second-line chemotherapy obtaining a PFS rate of 4 months. Patient 7 (a 65-year-old, never-smoker female, initial stage: cT1bN1M1a) started palliative chemotherapy with pembrolizumab and pemetrexed/carboplatin after droplet digital PCR–based *EGFR* mutation test (GenesWell ddEGFR Mutation Test) of the metastatic tumor in the interlobar lymph revealed no *EGFR* mutation in exons 18 to 21. The treatment strategy was determined based on the PCR-based assay. However, 1 month after the initiation of treatment, NGS examination of the same biopsy specimen detected *EGFR* L833V/H835L mutations, and the regimen was changed to afatinib (30 mg/once daily instead of 40 mg/once daily because of severe diarrhea). Four months after the administration of the afatinib, disease progressed, and PCR-based *EGFR* mutation test of the newly obtained biopsy specimen revealed no *EGFR* mutation, including the *EGFR* resistance mutation. The regimen was changed to pemetrexed/carboplatin, but the patient expired after 2 months.

### *EGFR* L833V/H835L cis mutant is oncogenic

**Figure 2.**
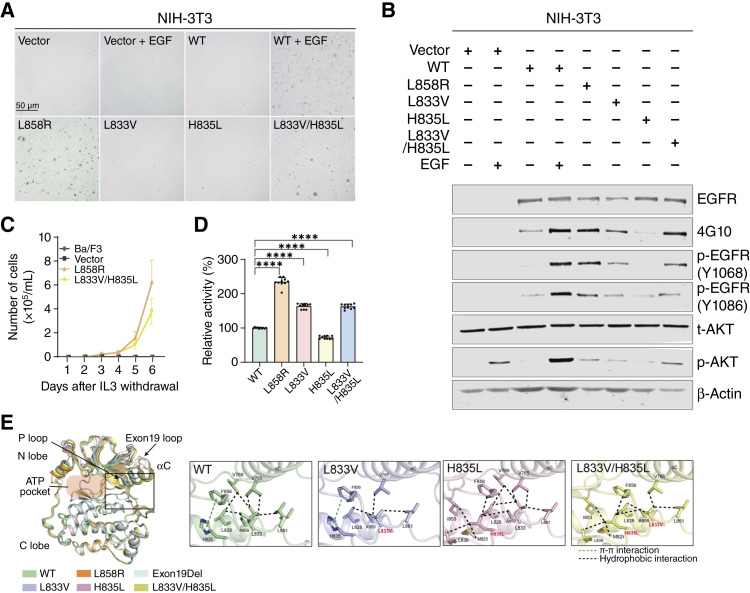
The *EGFR* L833V/H835L mutant is oncogenic *in vitro*. **A,** NIH-3T3 cells encoding WT or mutant EGFR were assayed for anchorage-independent growth in soft agar with or without 25 ng/mL EGF stimulation. Representative photomicrographs of soft agar colonies from three independents results are shown. **B,** Cell lysates prepared from NIH-3T3 cells expressing the indicated oncogenic EGFR mutants or WT EGFR were analyzed by immunoblotting with antibodies against t-EGFR, p-EGFR, t-AKT, and p-AKT with β-actin. **C,** Cell proliferation ability of various transformed Ba/F3 cell lines used for panel was assayed by counting cell numbers on 1 to 7 days later (0.2 × 10^6^ cells/mL each cell lines were seeded on day 0 after 2 weeks of IL3 withdrawal). The graph is indicated as mean with four independent counts each mutants (mean ± SD). **D,***In vitro* kinase activity of EGFR kinase variants bearing indicated mutations. Relative activity was normalized to WT, which was set to 100%. Bars represent the mean ± SEM (*n* = 10), ****, *P* < 0.0001 vs. WT (one-way ANOVA). **E,** Overall structural alignment of EGFR kinase domains and detailed view of interface between the αC-helix and activation loop. Labels for mutated residues are shown in red. Hydrophobic interactions are indicated by black dashed lines, and π–π interactions are shown as green dashed lines.

To investigate the oncogenic potential of these rare *EGFR* mutations, we engineered NIH-3T3 cells to stably express either WT or mutant *EGFR* (L833V, H835L, L833V/H835L, and L858R) through retroviral infection. Subsequently, we assessed the anchorage-independent growth of these established cell lines in soft agar, a hallmark of cellular transformation (Fig. 2A). NIH-3T3 cells expressing either the empty vector, WT *EGFR*, or the L833V or H835L single mutants did not form visible colonies in soft agar. Conversely, the L833V/H835L mutant expressing cells formed colonies, which are comparable in both size and numbers with those induced by the oncogenic L858R mutant in the absence of ligand stimulation. Consistent with its functional consequence, immunoblotting analysis revealed that the level of constitutive phosphorylation of L833V/H835L-mutant *EGFR* is more robust than that of single mutants, which is equivalent to those observed in oncogenic L858R mutant (Fig. 2B). In contrast, the phosphorylation is either absent in vector control or only observed in WT EGFR-expressing cells when stimulated with EGF. In addition, the phosphorylation levels of AKT, one of key downstream effectors of EGFR signaling pathway, are more pronounced in cells expressing both the L858R and L833V/H835L mutants compared with cells expressing single mutants, suggesting that the L833V/H835L mutant is constitutively active, similar to the oncogenic L858R mutant. Moreover, the L833V/H835L mutant enabled Ba/F3 cells to proliferate independently of IL3, an oncogenic property shared with the L858R mutant, providing strong evidence that the L833V/H835L mutant possesses oncogenic potential equivalent to that of the L858R mutant (Fig. 2C).

Next, we explore the impact of the L833V and H835L mutations on EGFR kinase activity using *in vitro* kinase assays (Fig. 2D; Supplementary Fig. S1A and S1B). Consistent with levels of phosphorylation observed in immunoblotting analysis, both the L833V and L833V/H835L mutants exhibited approximately twofold higher activity compared with WT EGFR. In contrast, the H835L mutant showed reduced activity, indicating that the L833V substitution, but not H835L, significantly enhances the intrinsic kinase activity of EGFR *in vitro*. To understand the structural consequences of these mutations, we compared apo kinase domain structures of WT and mutant *EGFR* (Fig. 2E). Structures of mutant *EGFR* were predicted using AlphaFold3, whereas experimentally determined structures were used for WT (PDB 7SI1), L858R (PDB 2EB3), and the Ex19del mutant (PDB 7TVD). The kinase C-lobe structure remained largely conserved across all variants. In contrast, the N-lobe exhibited notable shifts, particularly in the αC-helix and exon 19 loop regions. Local structural interactions were further analyzed to elucidate how these mutations alter kinase regulation (Fig. 2E; Supplementary Fig. S2A). In the WT structure, L833 forms strong hydrophobic contacts with V765 and V769 in the αC-helix, whereas H835 engages in a weak π–π interaction with F856, a key residue of the regulatory spine (R-spine) within DFG motif (22).

In the L833V mutant, the contact with V769 is lost, resulting in reduced anchoring of the αC-helix, whereas the H835–F856 interaction remains unchanged. In the H835L mutant, the L833–αC-helix contact is preserved, and residue substitution at H835 generates additional hydrophobic packing. The L833V/H835L double mutant displayed a mixed pattern in which the weakened L833–αC-helix interaction coexists with a localized hydrophobic cluster. Structural alignment further showed that this combination leads to an outward displacement of the αC-helix (Fig. 2E; Supplementary Fig. S2B and S2C). In addition, R-spine residues and αC-helix residues V765 and I759, exhibited positional shifts that correlated with the degree of αC-helix displacement. These structural rearrangements provide a mechanistic basis for the enhanced oncogenic potential of the L833V/H835L double mutant, as the outward αC-helix movement and altered R-spine alignment likely facilitate a more active kinase conformation, consistent with the phosphorylation, AKT activation, and transformation phenotypes observed in our cellular assays.

### L833V/H835L mutant is sensitive to EGFR inhibitors *in vitro* and *in vivo*

**Figure 3.**
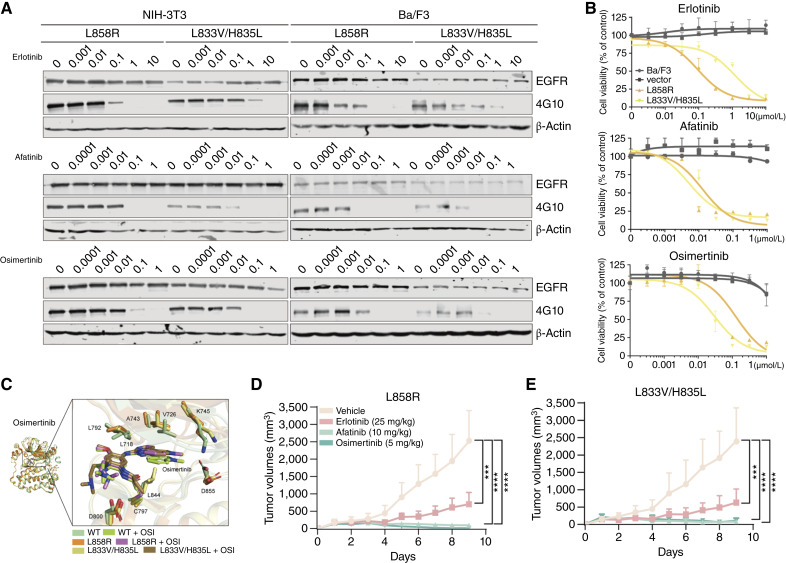
The *EGFR* L833V/H835L mutant exhibits sensitivity to EGFR TKIs both *in vitro* and *in vivo*. **A** and **B,** NIH-3T3 (**A**) or Ba/F3 (**B**) cells expressing the indicated *EGFR* mutants were treated with erlotinib, afatinib, or osimertinib at the concentrations indicated for 6 hours, and the resulting lysates were subjected immunoblotting with against phospho-tyrosine (4G10) or EGFR with β-actin as a loading control. **C,** Structural alignment and ligand-binding environment of the predicted osimertinib–L833V/H835L complex with experimentally determined structures of the WT and L858R EGFR–osimertinib complexes. **D** and **E,** The xenograft mouse models were generated by subcutaneous injections of NIH-3T3 cells expressed L858R or L833V/H835L mutations. When mouse tumors reached size of 150 mm^3^, erlotinib, afatinib, or osimertinib was administered by oral gavage. Tumor size was measured daily, and the volume was determined according to the formula V = AB^2^/2, in which **A** and **B** were tumor length and width, respectively (*n* = 9–10 for each treatment group, mean ± SD; ***, *P* < 0.001; and ****, *P* < 0.0001).

Next, we assessed the sensitivity of the L833V/H835L *EGFR* mutant to varying doses of EGFR-targeted drugs, including erlotinib, afatinib, and osimertinib, by measuring its phosphorylation via immunoblotting in NIH-3T3 and Ba/F3 cells and comparing it with the L858R mutant (Fig. 3A). Afatinib and osimertinib treatment significantly suppressed EGFR phosphorylation in L833V/H835L mutant cells (both NIH-3T3 and Ba/F3 cells) in a dose-dependent manner, exhibiting a more pronounced effect than observed in L858R-expressing cells. Conversely, erlotinib showed the opposite effect when comparing L858R and L833V/H835L mutants. Furthermore, we observed all three drugs dose-dependently reduced the viability of Ba/F3 cells transformed by these mutants (Fig. 3B). Ba/F3 cells expressing the L833V/H835L mutant demonstrated diminished sensitivity to erlotinib compared with L858R mutant–expressing cells. In contrast, treatment with afatinib and osimertinib led to comparable or enhanced reductions in cell viability for the L833V/H835L mutant relative to the L858R mutant, respectively. These observed differential sensitivities were further substantiated by the IC_50_ values determined for the *EGFR* mutants against the three tested drugs (Supplementary Fig. S1C). Specifically, the L833V/H835L mutant exhibited the lowest IC_50_ value of 6.84 nmol/L in response to osimertinib, underscoring its effective sensitivity to this particular inhibitor.

To further explore the structural basis for the increased sensitivity to osimertinib in the L833V/H835L mutant, we performed structural alignment of osimertinib-bound EGFR complexes—WT (PDB 6JXT), L858R (PDB 6JWL), and the AlphaFold3-based docking model of the L833V/H835L mutant (Fig. 3C). In the L833V/H835L mutant model, osimertinib binds adjacent to Cys797, where it forms a covalent bond consistent with its known mechanism of irreversible inhibition. The orientation of the ligand and surrounding interaction residues were conserved across all three models. Similar binding poses were also observed for erlotinib and afatinib (Supplementary Fig. S2E) within the ATP-binding pocket. Additionally, the overall structure of the inhibitor-bound models closely resembled the WT EGFR conformation, with no significant conformational deviations observed in the binding site. These findings suggest that the differential drug response observed in functional assays is not driven by altered inhibitor binding geometry but rather by mutation-induced shifts in the basal conformation and regulatory dynamics of the kinase domain, which may render the L833V/H835L mutant more susceptible to irreversible inhibition.

Next, we investigated the therapeutic efficacy against tumors harboring the L833V/H835L mutant with xenograft mouse models, which were established using NIH-3T3 cells transformed with expression of either the L858R or L833V/H835L mutant (Fig. 3D and E; Supplementary Fig. S3A and S3B). Subsequently, the antitumor responses to the drugs were assessed by monitoring tumor sizes in these mouse models. Consistent with our *in vitro* findings, both L858R and L833V/H835L mutants formed tumors in the xenografted mice, further confirming its oncogenic potential. Treatment with all three drugs effectively reduced the tumor formation in both the L858R and L833V/H835L xenograft mouse groups. Notably, more significant responses were observed in groups treated with either afatinib or osimertinib than erlotinib, as shown in *in vitro* experiments, suggesting that tumors harboring the L833V/H835L mutant are more sensitive to EGFR inhibitors, especially irreversible EGFR inhibitors.

## Discussion

To the best of our knowledge, the current study represents the largest investigation into NSCLC harboring either the *EGFR* L833V or H835L mutation with histopathologic analysis. To date, only 13 cases of the *EGFR* L833V/H835L mutations with documented clinical information have been reported, none of which included surgically resected specimens or detailed histopathologic features (Supplementary Table S1).

Our findings indicate that this mutation is frequently associated with poorly differentiated histology. Total five cases with the *EGFR* L833V/H835L in cis mutation received histopathologic examination after resection, which revealed 20% or more of either the micropapillary or solid pattern (hence, poorly differentiated histology) in four of five resected cases (Fig. 1C–F). Micropapillary and solid patterns in adenocarcinoma are known indicators of worse prognosis (23). A study of 198 resected lung adenocarcinomas from East Asian, female never-smokers found micropapillary and solid patterns in 18.2% and 16.7%, respectively (2).

Our findings indicate that this mutation is frequently associated with advanced disease presentation. In the present cases, all 11 patients with either *EGFR* L833V or H835L mutation presented with adenocarcinoma. Notably, 10 of these 11 patients were diagnosed at stage III or IV, and the remaining patient (patient 8, initially pT2aN0M0) developed pleural metastasis for 2 months after surgical resection (Table 1). Kim and colleagues (3) reported that patients with compound *EGFR* mutation showed poorer clinical outcomes than those with single classic mutations.

Our findings indicate that *EGFR* L833V/H835L cis mutations are not only exceptionally rare but also biologically oncogenic. Our findings indicate that the *EGFR* L833V mutant, despite demonstrating elevated basal kinase activity and receptor autophosphorylation, did not demonstrate sufficient oncogenicity to transform NIH3T3 and Ba/F3 cells in this study, which is in contrast with a previously published report (24). Conversely, the L833V/H835L mutant clearly displayed oncogenic properties, successfully transforming both cell lines. This outcome suggests that whereas individual *EGFR* L833V and H835L mutations may possess limited or relatively less oncogenic activity, their co-occurrence in cis creates a synergistic effect, significantly enhancing oncogenic potential, consequently activating oncogenic signaling pathways like AKT. Our structural modeling analysis revealed that L833V disrupts hydrophobic contacts that anchor the αC-helix, thereby increasing structural flexibility and catalytic activity, whereas H835L forms a hydrophobic core that restricts helical mobility and inhibits activation. Notably, the L833V/H835L double mutant displayed elevated kinase activity comparable with L833V, suggesting that the activating effect of L833V predominates over the stabilizing influence of H835L. Structural modeling revealed that the double mutant exhibits a more pronounced outward displacement of the αC-helix, which stabilizes the asymmetric dimer and likely enhances receptor dimerization and oncogenic signaling. Consistent with this, dimer interface analysis showed that the L833V/H835L mutant has an increased surface contact area in both the activator and receiver kinases (Supplementary Fig. S2D and S2E). Such structural alterations may promote dimerization and modulate signaling output in cellular contexts, helping to explain the relationship between monomer-based *in vitro* kinase assay results and cellular oncogenic activity.

Our findings in the *in vitro*, *in vivo*, and structure analysis data support the use of third-generation EGFR TKIs for the *EGFR* L833V/H835L cis mutation, although four patients in the clinical cases with the *EGFR* L833V/H835L in cis mutation were treated with early-generation EGFR TKIs, none of which were the first-line therapy. As the turnaround time of NGS test is longer than that of PCR-based assays, initial treatment strategy was often determined based on the result of the PCR-based assays, all of which used in this study could not identify this rare compound mutation. After detection of the mutation via NGS test, still, use of early-generation TKIs was preferred as the selection of treatment is heavily influenced by the South Korea’s unique national health insurance reimbursement system, which is mandatory and prioritizing the common sensitizing classic mutation. However, real-world data reporting response to third-generation EGFR TKIs have been emerging recently (Supplementary Table S1; refs. 15, 16).

### Conclusions

Our *in vitro* and *in vivo* xenograft models demonstrate the enhanced sensitivity of the *EGFR* L833V/H835L mutant to second- and third-generation EGFR inhibitors. This finding is particularly significant given the absence of established clinical guidelines for this rare mutation, thus providing a crucial therapeutic direction. Our study highlights the importance of evaluating compound *EGFR* mutations which are not captured by some commercial PCR-based assays, as they may confer distinct biological behaviors and potential actionable target.

## Supplementary Material

Table S1 Supplementary Table 1

Figure S1 Fig. S1. Mutational impact on EGFR kinase structure, R-spine alignment, and dimer interface

Figure S2 Figure S2. Purification profiles and in vitro kinase activity of WT and mutated EGFR kinases.

Figure S3 Figure S3. Response of EGFR-TKI in xenograft mouse model

## Data Availability

All relevant data are available from the authors upon request.
