# Supplementary figures and images for: Clinical and Molecular Characterization of a Rare EGFR cis Compound L833V/H835L Mutation in Non–Small Cell Lung Cancer
Source: Cancer Res Commun. 2026 Apr 22;6(4):913–22. doi: 10.1158/2767-9764.CRC-25-0831 (PMC13101886; doi:10.1158/2767-9764.CRC-25-0831)

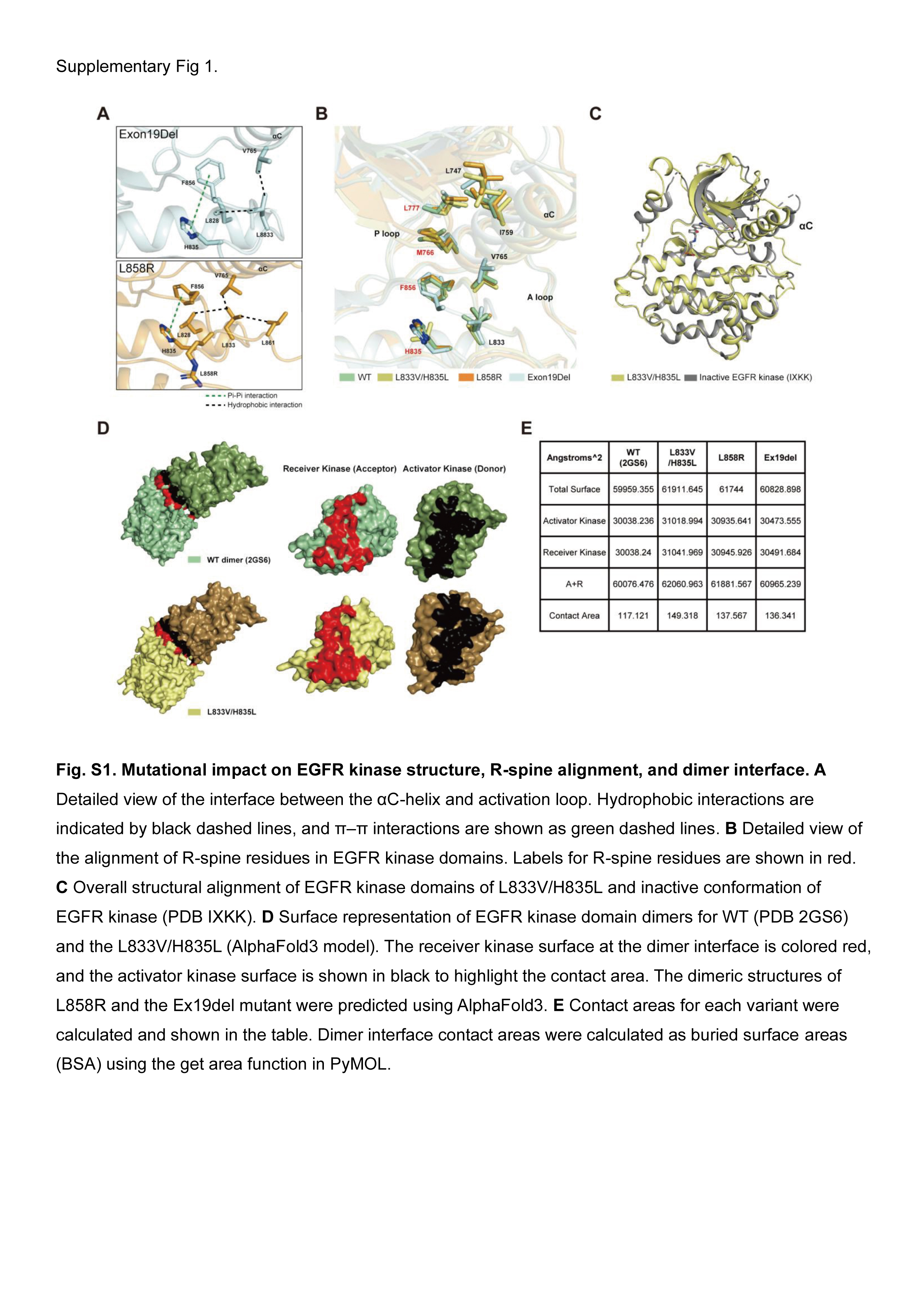

Supplement: Figure S1 — Fig. S1. Mutational impact on EGFR kinase structure, R-spine alignment, and dimer interface [file crc-25-0831_figure_s1_suppsf1.jpeg]

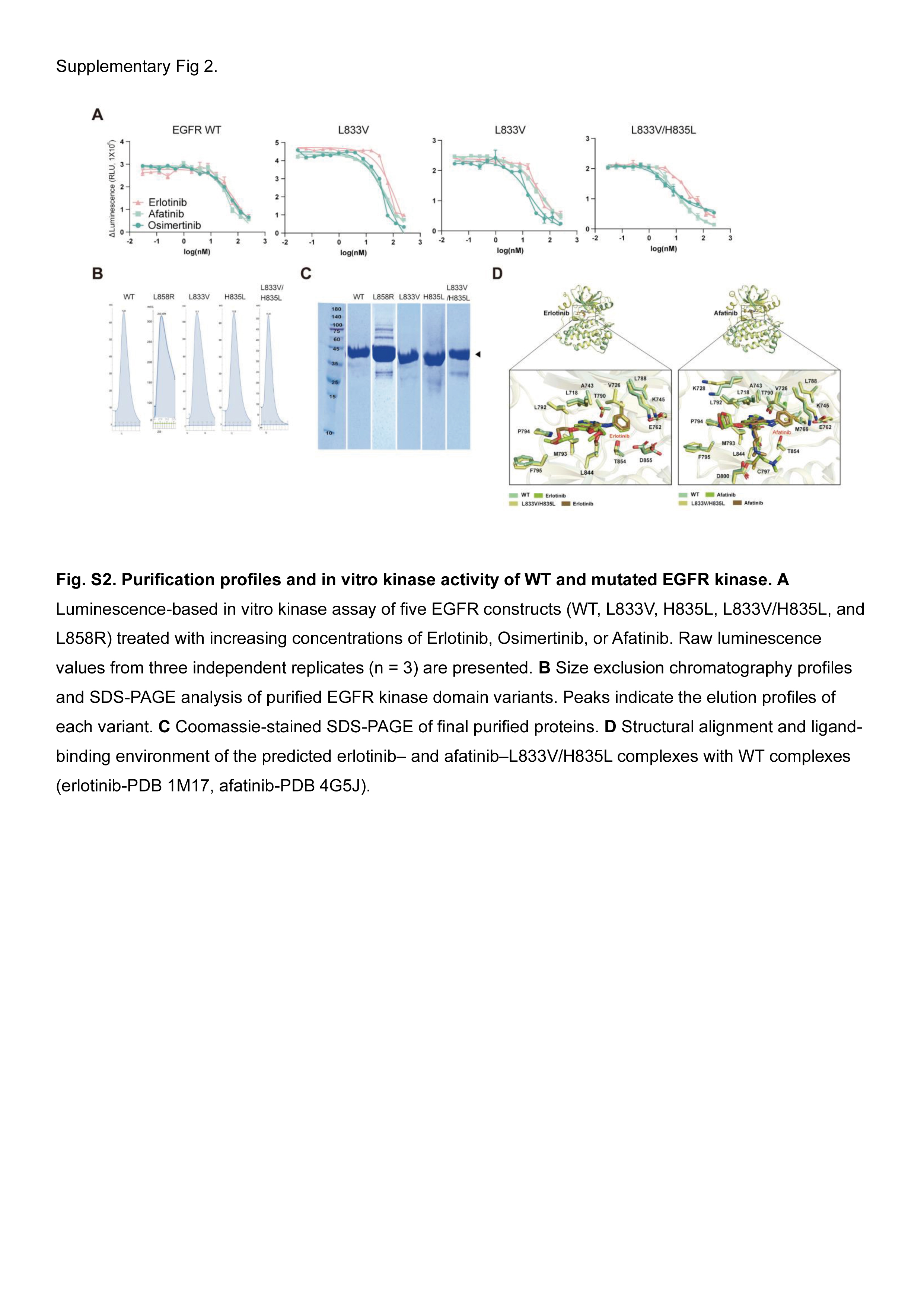

Supplement: Figure S2 — Purification profiles and in vitro kinase activity of WT and mutated EGFR kinases. [file crc-25-0831_figure_s2_suppsf2.jpeg]

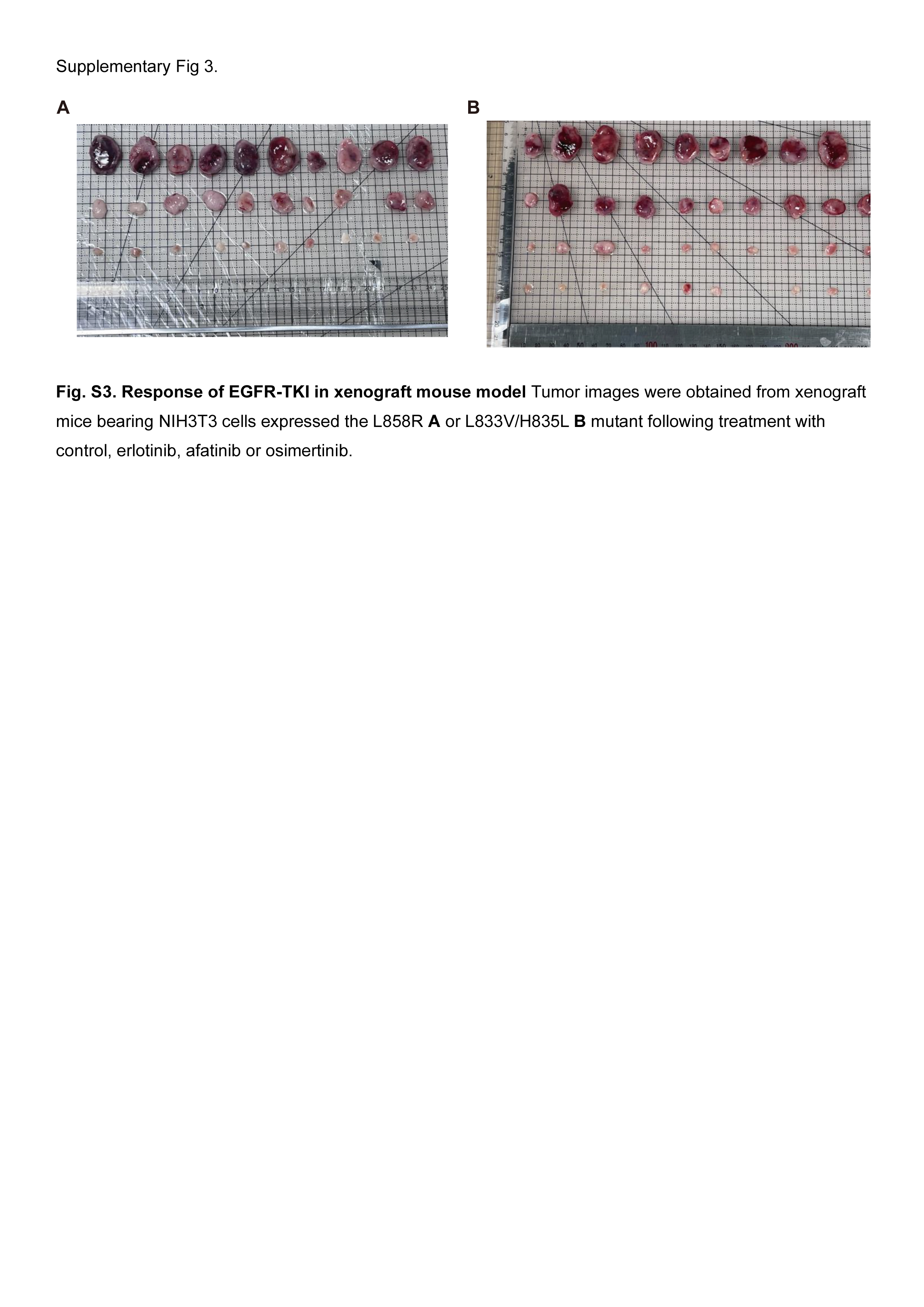

Supplement: Figure S3 — Response of EGFR-TKI in xenograft mouse model [file crc-25-0831_figure_s3_suppsf3.jpeg]
